# Modified Clagett Procedure Is Effective for Methicillin-Resistant Staphylococcus aureus Postpneumonectomy Empyema: A Case Report

**DOI:** 10.14740/wjon822w

**Published:** 2014-08-25

**Authors:** Stefan Welter, Sandra Kampe, Jan Dziobaka, Eleftherios Chalvatzoulis, Mahmood Zahin, Christian Roesel, Georgios Stamatis

**Affiliations:** a Department of Thoracic Surgery and Thoracic Endoscopy, Ruhrlandklinik, West-German Lung Center, University Hospital, University of Duisburg-Essen, Germany; b Department of Anesthesiology, Ruhrlandklinik, West-German Lung Center, University Hospital, University of Duisburg-Essen, Germany; c Institute of Medical Microbiology, University Hospital Essen, University of Duisburg-Essen, Essen, Germany

**Keywords:** MRSA, Complication, Postpneumonectomy empyema

## Abstract

Postpneumonectomy empyema (PPE) with methicillin-resistant *Staphylococcus aureus* (MRSA) is a challenging problem because these germs have extensive virulence factors and mechanisms to escape from the host’s immune system. The present case was successfully treated with accelerated repeated surgical debridement, vancomycin gauze packing and final obliteration of the postpneumonectomy space with latissimus myoplasty and vancomycin solution.

## Introduction

Postpneumonectomy empyema (PPE) is a serious complication after major surgery for lung malignancies, with a mortality of 25-50% [1]. There are three important treatment steps: rapid and effective drainage, assessment of bronchopleural fistula (BPF) and obliteration of the pleural cavity [2]. Various treatment strategies have been described. One involves surgical debridement and fistula closure if present, followed by continuous antibiotic irrigation until cultures are negative [3]. This strategy may require a mean of 40 days irrigation time.

A second strategy involves accelerated treatment with open debridement and lavage followed by second-look operations every second day. In one study, this strategy was effective in 75% of patients [4]. A third strategy is empyema debridement by open window thoracostomy (OWT) followed by later obliteration of the cavity with thoracoplasty and myoplasty procedures [2]. Serious problems may arise when methicillin-resistant *Staphylococcus aureus* (MRSA) is responsible for the empyema, because MRSA is well known to persist in chronic wounds and empyema cavities [5]. Successful antibiotic-irrigation with arbekacin of empyema cavities was only reported in some cases after non-pneumonectomy lung resections [6]. We report successful MRSA eradication from a postpneumonectomy space by the use of aggressive open debridement, intracavitary use of vancomycin and a latissimus dorsi muscle myoplasty.

## Case Report

We report the case of a 58-year-old man (50 kg body weight) with a right-sided MRSA PPE. He was readmitted to the intensive care unit critically ill with severe dyspnoea, fever (maximum 42 °C), pancytopenia and coagulopathy. Fourteen weeks earlier he underwent extended right pneumonectomy with partial resection of the left atrium, resection of the pericardium and Vicryl mesh coverage. Three days before readmission the second cycle of adjuvant chemotherapy (doxorubicin and ifosfamide) was applied. The R0 lung resection had been performed in April 2013 for a central pulmonary high grade sarcoma (NOS) measuring 15.3 × 13.3 × 10.0 cm and involving the right middle and lower lobe. The bronchial stump had been covered with a pericardial fat pat.

**Figure 1:**
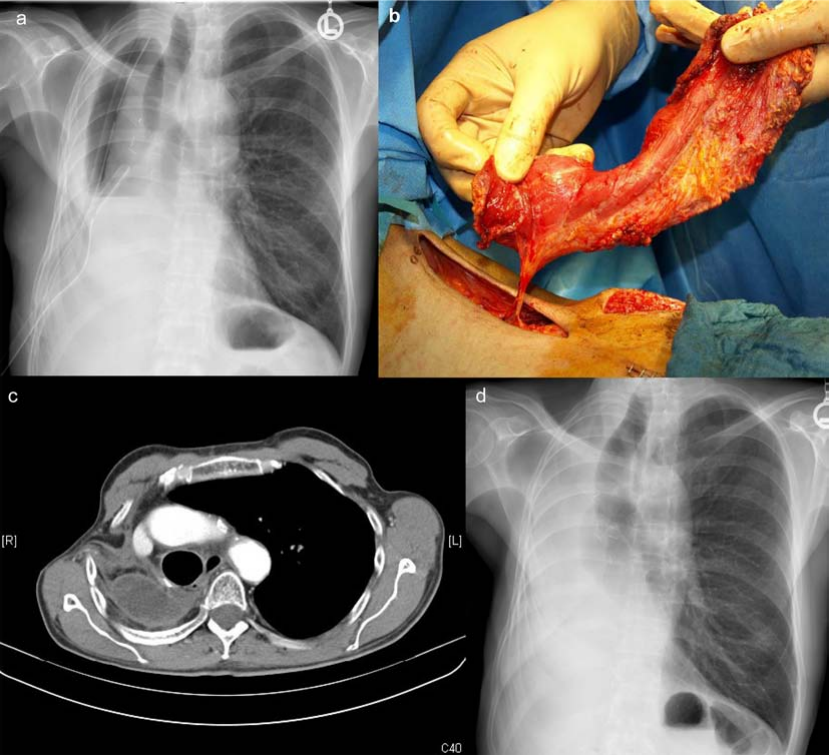
Images demonstrating the progress of treatment: (a) X-ray of the chest demonstrating thickened right-sided thoracic wall and two drains, the day before open debridement; (b) preparation of the pedicled latissimus dorsi muscle flap; (c) CT-scan 3 months after definitive closure demonstrating adequate shift of the mediastinum to the right side and the entrance of the muscle; (d) corresponding chest X-ray after 3 months.

After readmission the patient was stabilized with intravenous (IV) antibiotics (meropenem 500 mg td, and vancomycin 500 mg bd) and thoracoscopic empyema debridement with the introduction of two drains, followed by daily irrigation with 1.5 L of normal saline. MRSA was isolated in the pus as well as in the nose and bronchial system. The calculated antibiotics were deescalated to IV vancomycin given 1 g bd. After 3 weeks MRSA still persisted in the irrigation fluid, and thus 1 g of vancomycin was instilled into the cavity after each irrigation. Seven days later MRSA still persisted in the fluid and thus the treatment strategy was changed (Fig. 1a). Open re-debridement with extensive washing with hydrogen peroxide and gauze packing with vancomycin-solution was performed three times every third day, combined with a latissimus muscle myoplasty (Fig. 1b) to cover the pericardial mesh and bronchial stump region as well as to reduce the residual space. The muscle was attached to the mediastinum with several polydioxanone sutures and fixed with another layer of antibiotic gauze. Wound closure with removal of the gauze and instillation of another 2 g of vancomycin took place 9 days after the first open debridement. The final microbiology was negative. The treatment was accompanied by superficial measures to eradicate MRSA from the skin, throat and nose.

IV vancomycin and intensive irrigation with normal saline did not eradicate MRSA, but allowed the patient to recover from severe septicemia. Filling the cavity with vancomycin solution was not effective either. The irrigation fluid remained cloudy and positive for MRSA (Fig. 1a). The open debridement demonstrated a thick peel formation with fibrin and pus attached all over the cavity. Two further debridements were necessary to gain adequate clearance before muscle coverage was possible (Fig. 1b). The patient tolerated all operative procedures and recovered, gained weight and was fully mobilized. He was discharged 17 days after the latissimus myoplasty. The pleural fluid aspirate 3 weeks and also 3 months later as well as the nose swabs and sputum were negative for MRSA, and the patient was free of inflammatory signs. Computed tomography of the thorax demonstrated no abscess formation and no signs of tumor recurrence at 3 months (Fig. 1c, d).

## Discussion

To our knowledge the treatment of MRSA in the postpneumonectomy space has only been described in a few non-English case reports and lacks the experience of large series. MRSA eradication from the skin is difficult and is almost impossible from chronic wounds without aggressive therapy regimens. Typical MRSA virulence factors such as surface proteins G and X (SasG, SasX), α- and β-toxin, clumping factor B, phenol-soluble modulins (PSMs) and leucotoxins lead to different degrees of colonization, invasion and dissemination within the host. The ability to produce a biofilm protects *Staphylococcus aureus* from host immune defenses and from antibiotics, leading to difficulties eradicating MRSA from postpneumonectomy spaces [5]. Our patient was readmitted when critically ill and was suffering from septicemia as well as the side effects of chemotherapy. Thoracoscopic debridement allowed the patient to recover from septicemia but did not eradicate MRSA from the postpneumonectomy space. Furthermore we found thick layers of clotted fibrin and pus all over the space despite the fact that drainage and daily irrigation were performed. This was consistent with the persistent signs of active inflammation seen in the blood results, and may explain why the administration of local antibiotics after 3 weeks of daily irrigation could not eliminate MRSA. Immediate debridement with OWT with antibiotic wound dressings, as described by others [1, 2] might have been more effective at controlling sepsis and eliminating MRSA compared with irrigation alone. However, OWT is associated with other long-term complaints that we always try to avoid.

Once we recognized that irrigation alone is not effective, we started an accelerated treatment similar to that described by Wojcik et al [4]. We are convinced that repeated aggressive debridement supplemented with antibiotic gauze packing was the key to successful MRSA eradication. Furthermore, the obliteration of the residual space with antibiotic solution prevented infection recurrence. Our decision to conduct a latissimus muscle myoplasty was based on the concern that microorganisms will persist in the Vicryl mesh on the heart and that direct contact with viable muscle would enable the immune system to cope with that localized problem.

### Conclusion

VATS debridement, daily irrigation and IV antibiotics were not sufficient to eradicate MRSA from the postpneumonectomy space. Aggressive surgical debridement, vancomycin gauze packing, the use of a viable muscle flap and the final obliteration of the residual space with antibiotic solution allowed enduring MRSA eradication in the presented case without the use of OWT.
